# Long-term use of cannabis-based medicines in two children with Tourette syndrome: a case report

**DOI:** 10.3389/fpsyt.2025.1647969

**Published:** 2025-10-02

**Authors:** Lara-Katharina Woerner, Natalia Szejko, Carolin Fremer, Simon Schmitt, Kirsten R. Müller Vahl

**Affiliations:** ^1^ Clinic of Psychiatry, Social Psychiatry and Psychotherapy, Hannover Medical School, Hannover, Germany; ^2^ Department of Bioethics, Medical University of Warsaw, Warsaw, Poland

**Keywords:** Tourette-syndrome, medicinal cannabis, children, long-term treatment, case report

## Abstract

**Introduction:**

Cannabis-based medicine (CBM) is recommended for the treatment of tics in otherwise treatment-resistant adult patients with Tourette syndrome (TS). However, evidence in children with TS is very limited. Long-term effects of CBM in this population are unknown.

**Case presentations:**

We present two cases of long-term follow-up over six and five years, respectively, in male adolescents with TS who were administered CBM starting at the age of eight and 12 years, respectively. In one patient CBM treatment was initiated with pure tetrahydrocannabinol (THC) and was later changed to current treatment with an oral THC-dominant cannabis extract (THC:cannabidiol (CBD)=25:<0.5) with a daily dose of 0.5-0.6 mL (corresponding to 12.5–15 mg THC/day). The other patient was from the beginning up to now medicated by his parents, who are physicians, with vaporized THC-dominant (24%) medicinal cannabis flowers with a dose of 0.2 g between once to thrice per day (corresponding to 48–144 mg THC/day). While in one patient, there was a moderate dose increase over the years, in the other patient dosages were adjusted individually depending on tic severity. In both patients, CBM treatment resulted in continued benefit with significant improvement of tics and psychiatric comorbidities without severe adverse effects. Academic performance of both adolescents was excellent. Neurocognitive assessments demonstrated average results in the domain of working memory and average to above average results in the domain of processing speed.

**Conclusions:**

We present two cases of minors with TS who started CBM treatment at the age of eight and 12 years, respectively, and continued treatment for five to six years resulting in clinically relevant symptom improvement without any severe adverse effects or negative impact on cognitive and academic performance.

## Introduction

Tourette syndrome (TS) is a childhood-onset neurodevelopmental disorder characterized by the presence of multiple motor and at least one vocal tic with a minimal duration of one year (1). On average, tics start at the age of five to seven years with peak severity occurring between the age of 10 to 12 years (2, 3). According to the American Academy of Neurology (AAN) (4) and the European Society for the Study of Tourette Syndrome (ESSTS) (5) the first-line therapy for tics include behavioral therapy and/or pharmacotherapy with antipsychotics or alpha-agonists. In otherwise treatment-refractory cases, only limited alternative treatment options are available including cannabis-based medicine (CBM) (5).

While there is an increasing evidence that CBM reduces tics in adults with TS (6–15), evidence in children and adolescents is very limited (16–19). Only recently, the first large randomized controlled trial (N = 97) investigating efficacy and safety of the cannabis extract nabiximols in adult patients with TS provided evidence for its effectiveness in this population, although the primary endpoint was narrowly missed (15). Interestingly, additional exploratory subgroup analyses revealed a larger improvement of tics in males, patients with more severe tics, and patients with comorbid attention deficit/hyperactivity disorder (ADHD) suggesting that these subgroups may benefit better from CBM treatment. There were no relevant safety issues.

In contrast, in children and adolescents with TS, so far only five case reports are available including a total of six patients as well as preliminary data from a small phase I/II double-blind, cross-over pilot study including ten patients, all describing *acute* effects of CBM and one study using real world data from teenagers and adults. The first case study was published as early as 1988 and reported two adolescents aged 15 and 17, who self-medicated with inhaled cannabis and experienced an improvement of tics of about 50% and comorbid symptoms including obsessive-compulsive disorder (OCD), self-injurious behavior (SIB), and attention problems (20). Twenty-two years later, in 2010, the first case of a treatment-resistant 15-year-old adolescent with TS and co-existing ADHD was published, whose symptoms significantly improved after treatment with pure tetrahydrocannabinol (THC, maximum dose = 15 mg THC/day) (16). Over a period of nine weeks, an improvement of tics and quality of life was clinically observed and measured before and after therapy with THC using standardized interviews, questionnaires and a video protocol (16).

More recently, our group reported three minors who benefitted significantly from treatment with different CBMs without any severe adverse effects (17–19). Jakubovski et al. (19) described a 16-year old adolescent with TS who struggled with a variety of speech problems such as palilalia, vocal blocking as well as change of melody and rhythm of speech. Since conventional therapy was not effective, treatment with oral THC (maximum dose = 33.6 mg THC/daily, later switched to vaporized THC-dominant medicinal cannabis flowers with a daily dose of 1.8 g) was started. At follow-up of up to eight months a marked reduction of motor and vocal tics including an improvement of speech fluency was reported by the patient. He further described an improvement of psychiatric symptoms such as impulsive behaviors, rage attacks, anxiety, and OCD and, in addition, reported no side effects due to the medication.

In 2025, results have been published from a double-blind, controlled pilot study in ten adolescents (mean age 14.8 years, range, 12–18 years, 50% male) performed in Australia (21). The primary aim was to investigate, whether the study protocol is feasible and acceptable using a CBM containing THC 10 mg/mL (depending on weight: max THC 10 mg/day, if < 50 kg or max THC 20 mg/day, > 50 kg) and cannabidiol (CBD) 15 mg/mL over 10 weeks. Overall, CBM was well tolerated, 7/10 participants completed the study, 2/10 discontinued due to adverse events (one on CBM, one placebo). The most common adverse event was dizziness (67%).

In a real-setting study from Israel published in 2024, a mixed sample of patients was evaluated regarding beneficial effects of CBM (oily solution or cannabis flowers) (22). They also report a significant improvement in quality of life, employment status, reduction in the number of other medications as well as improvements in OCD and anxiety. However, follow-up lasted only 6 months. Since only 7 out of 70 patients were minors and these results were not reported separately, clinical effects specifically in this subgroup remain unclear.

Alongside the limited amount of data regarding the use of CBM in children, there are also some reports in other neuropsychiatric disorders in pediatric populations, mainly in autism spectrum disorder (ASD) where CBD can improve behavioral problems, anxiety, and sleep (23–26). According to a recently published systematic review of the very limited number of RCTs in minors, a modest positive effect of CBM in ASD has been found (26).

Here, we report tic severity and commonly associated comorbidities such as ADHD, OCD, and depression as well as overall performance at long-term follow-up of two cases published recently by our group (17, 18). Both cases are still the only available reports describing *acute* effects of CBM in children with TS.

While this limited number of case studies suggests that CBM may be useful for the *acute* management of tics – and possibly also psychiatric comorbidities - in minors with TS, we do not have any information about efficacy and, in particular, safety of this treatment as continuous medication. This is of great relevance since there are general concerns regarding repercussions of CBM treatment in children (27). The main considerations are related to the risk of psychiatric side effects and a negative impact on academic performance as well as cognitive functioning (28–31). In this study, we therefore also conducted assessments of cognitive domains such as processing and working speed. Furthermore, we analyzed school reports to assess academic functioning. All tests were either self-administered or administered by clinician and researchers with experience in tics. For both cases, patients and parents provided written informed consent for publication of the cases (please refer to the Statement of Ethics section). For treatments as part of an individual therapeutic trial, no ethical approval is required in Germany.

## Case descriptions

### Case 1

Case 1 was published in 2018 by Szejko et al. (17) and describes a boy diagnosed with TS and comorbid ADHD at age 6. At age 7, his tics deteriorated and markedly compromised his quality of life. Since his ability to write and his school performance were significantly impaired, he finally refused to attend school leading to social isolation, depression, social anxiety, separation anxiety, and suicidal thoughts. After several different treatments (pharmacological therapy and psychotherapy) for tics and ADHD had failed to improve his symptoms, treatment with oral THC was initiated, when he was eight years old. At a dose of 18 mg THC/day, his tics improved by 50%. At the same time, the patient as well as his parents and teachers reported an improvement of ADHD symptoms, overall behavior, school performance, and resumption of social activities and interests, resulting in an enormous improvement of his quality of life. No side effects occurred. For further details please refer to (17).

Over the following three years, he was treated with oral THC in varying doses (on average: 16.0 mg THC/day, minimum dose: 8.0 mg THC/day, temporally maximum dose: 29.4 mg THC/day, with about 2/3 of dose in the morning before school and 1/3 in the afternoon). While treatment with guanfacine (2 mg/day) for ADHD was continued, treatment with risperidone (1 mg/d) for tics was discontinued 4 months after initiation of CBM. At age 10, his tics increased despite increased THC dose up to 26 mg THC/day, which might be related to the typical course of TS with a tic maximum at the age of 10–12 (2, 3), and, in addition, to quite stressful time at school since the patient repeated a grade. Further increase of THC was not possible due to side effects such as increased sedation. Therefore, we decided to switch from pure THC to an oral THC-dominant cannabis extract (THC: CBD = 25:<0.5). Under this treatment, motor and vocal tics significantly decreased with no severe adverse effects. For the following three years, he was treated with a constant dose of 0.5 mL of the oral cannabis extract twice daily corresponding to 25 mg THC/day. Only during school vacations and at weekends, parents temporarily reduced the dosage. In the following years, tics further improved and in parallel, dosage of the cannabis extract was reduced first to 0.5 mL – 0 – 0.3 mL per day (corresponding to 12.5 - 0 – 7.5 mg THC) and later even to 0.5 mL once daily. An interim trial with a cannabis extract containing higher dose of CBD (THC 25:CBD 25) did not lead to further improvement in his tics. At the time of the last follow-up, at age 16 – six years after initiation of CBM - the patient took between 0.2 mL and 0.4 mL of a THC dominant cannabis extract (THC: CBD = 25:<0.5, corresponding to 5–10 mg THC/d) once in the morning, adjusted to symptom severity.

As illustrated in **Table 1**, positive effects lasted over six years, which included reduction of tics according to the total tic score of the Yale Global Tic Severity Scale (YGTSS-TTS) (32) and depressive symptoms measured with the German Children’s Depression Inventory (Depressionsinventar für Kinder und Jugendliche, DIKJ (33)). Data showed also improvements of ADHD symptoms as assessed with the Swanson, Nolan and Pelham Teacher and Parent Rating (SNAP) (34) as well as reduction of perceived stress according to the Perceived Stress Scale (PSS-P) (35) and the Strengths and Difficulties Questionnaire (SDQ) (36), and improvement of quality of life according to Gilles de la Tourette Syndrome-Quality of Life Scale (GTS-QOL) (37) and Kinder-Lebensqualität, a German instrument to measure health-related life quality in children (KID-KINDL) (38). In contrast, premonitory urges (according to the Premonitory Urge for Tics Scale, PUTS) (39) increased compared to baseline and short-term follow-ups (17). Aside from mild sedation experienced at the beginning of treatment and at high doses of THC, no adverse effects occurred.

**Table 1:** Case 1: clinical measurements before and after long-term treatment with THC and THC-dominant cannabis extract.

Variable	Scale [range]	Baseline	Long term follow-up (6 years)	Change [%] baseline vs. follow up
Age		8y9m	14y8m	
Tics	YGTSS-TTS [0-50]	38	23	-39,47%
Premonitory urge	PUTS [0–40]	11	18	+63,64%
Impairment/ quality of life	YGTSS-Impairment Scale [0-50] GTS-QOL [0-100] GTS-QOL-VAS [0–100] KID-KINDL [0-100] CGI [0–7]	30 42 60 65 5	0 3 100 90 4	-100% -92,86% +66,67%* +38,46%* -20%
Tics + impairment	YGTSS – Global Score [0–100]	68	23	-66,18%
Depression Stress Behavior Autistic traits ADHD OCD	DIKJ [33-80, T-Wertbande] PSS-P [0-40] SDQ [0–40] ASSF [0-56] SNAP [0-54] CY-BOCS [0-40]	53 36 40 22 34 0	33 12 26 12 2 0	-37,74% -66,67% -35% -45,45% -94,12% 0%
Intelligence**	WISC [percentile ranks] Processing speed Working memory TMT Trail A Trail B	/ / / /	90% 34% 21.29s 44.10s	

*The higher the score, the better quality of life. **40-60% is considered normal result.

YGTSS-TTS, Total tic score of the Yale Global Tic Severity Scale; ATQ, Adult Tic Questionnaire; PUTS, Premonitory Urge for Tics Scale; GTS-QOL, Gilles de la Tourette Syndrome – Quality of Life Scale: higher results indicate worse quality of life; GTS-QOL-VAS, Visual Analogue Scale of Gilles de la Tourette Syndrome – Quality of Life Scale; higher results indicate better quality of life; CGI, ClinicalGlobal Impression Severity Scale; CGI-I, Clinical Global Impression –Improvement Scale; DIKJ, Depressionsinventar für Kinder und Jugendliche, German instrument to measure intensity of depression in children and adolescents; PSS-P, Perceived Stress Scale; SDQ, The Strengths and Difficulties Questionnaire; ASSF, Autismus-Spektrum Screening Fragebogen; SNAP, Swanson, Nolan and Pelham Teacher and Parent Rating; CY-BOCS, Children’s Yale-Brown Obsessive Compulsive Disorder [cut off: <=16]; WISC, Wechsler Intelligence Scale for children- fifth edition; TMT, Trail Making Test (Trail A: Deficient: >78 sec.; Trail B: >273 sec.).

To investigate cognitive performance, we administered Wechsler’s Intelligence Scale for Children (WISC) during the patient’s last visit (40). Only the domains “processing speed” and “working memory” were tested, as the literature repeatedly reports that use of cannabis may cause particularly impairments in these areas. The patient achieved a slightly below average result for the working memory domain and an above average result in the processing speed domain. The average results in the WISC score are considered to be between 40-60%, while for our patient it was 34% and 90% for working memory and processing speed, respectively.

Since the patient is attending a school where no grades are awarded, it is difficult to make a statement about measurable academic success. However, it can be stated that in the 5^th^ grade - when therapy with CBM was initiated - the patient predominantly received the assessment “good” to “satisfactory”. His behavior was also in line with social standards. In the 6^th^ grade, the boy became class president. In his 7^th^ grade report card, he received mostly “good” to “very good” ratings. At the end of the school year, he wants to graduate from secondary school and then plans to attend grammar school in order to obtain his High school degree (Abitur). His behavior at school fully met expectations and his social behavior deserved special recognition. He has friends and pursues hobbies. Furthermore, no symptoms of psychosis, anxiety, depression, addiction, or any other newly developed psychiatric symptoms occurred at any time during the treatment period. At no time there was evidence of cannabis use disorder (CUD) and additional use of recreational cannabis was explicitly denied.

### Case 2

Case 2 was published in 2019 by Szejko et al. (18). We reported a successful treatment of a 12-year-old boy, whose tics had started at age 6. At age 12, he presented for the first time at our clinic. At that time, his parents, who both were medical doctors, had already initiated treatment with CBM. A combined treatment with vaporized medicinal cannabis flowers (up to 0.1 g/day) and oral THC (maximum dose = 12.5 mg THC/day) resulted in a marked improvement of simple and complex motor and vocal tics, headaches, obsessive-compulsive behavior (OCB), sleeping problems, and mild self-injurious behavior. No side effects were reported (for further details please refer to (18)).

Here, we report a long-term follow-up of five years of CBM therapy. During this time period, CBM treatment was continued without withdrawal but with dose changes depending on tic severity. Treatment was supervised by his parents. At long-term follow-up, the patient was taking 0.2 g medicinal cannabis (Hindu-Kush or Gorilla Glue, both containing 24% THC) once to thrice per day (which corresponds to 48–144 mg of pure THC), with dosage adjusted as needed. Compared to initial assessments (18), the patient continues having positive effects on his tics and quality of life (see **Table 2**). With regard to his tics, there was a sustained improvement as measured by YGTSS-TTS (32) and ATQ. However, premonitory urges (as measured by PUTS (39) increased compared to baseline. At baseline, there was no evidence for psychiatric comorbidities including ADHD, depression, OCD, and ASD. Follow-up after five years showed no change in depressive symptoms as measured with DIKJ (33), ADHD as measured with Diagnostikfragebogen ADHS (ADHD-DQ) (34), stress and behavior according to the PSS-P (35), and improvement of quality of life according to GTS-QOL (37) and KID-KINDL (38). However, compared to baseline, the patient developed moderate OCD (according to Children’s Yale-Brown Obsessive Compulsive Disorder, CY-BOCS) (41).

**Table 2:** Case 2: clinical measurements before and after treatment with vaporized medicinal cannabis flowers.

Variable	Scale [range]	Baseline [before cannabis inhalation]	Long term follow up 5 years	Change [%] baseline vs. long-term follow up
Age		12y1m	16y11m	
Tics	YGTSS-TTS [0-50] ATQ [0-224]	29 131	24 103	-17,24% -21,37%
Premonitory urge	PUTS [0–40]	23	27	+17,39%
Impairment/ Quality of life	YGTSS-Impairment [0-50] GTS-QOL [0-100] GTS-QOL-VAS [0–100] KID-KINDL [0-100] CGI [0–7]	30 42 45 86 3	2 20 80 92 3	-93,33% -52,38% *+77,78% *+6,98% 0%
Tics + impairment	YGTSS Global Score [0–100]	59	26	-55,93%
Depression Stress Autistic traits ADHD OCD	DIKJ [33-80, T-score] PSS-P [0-40] ASSF [0-54] SNAP [0-54] ADHD-DQ CY-BOCS [0-40]	50 / 11 / 0 0	41 11 3 6 / 22	-18% / -72,73% / / /
Intelligence**	WISC [percentile ranks] Processing speed Working memory TMT Trail A Trail B	/ / / /	50% 34% 29:63 sec 105:38 sec	

*The higher the score, the better quality of life. **40-60% is considered normal result.

YGTSS-TTS, Total tic score of the Yale Global Tic Severity Scale; ATQ, Adult Tic Questionnaire; PUTS, Premonitory Urge for Tics Scale; GTS-QOL, Gilles de la Tourette Syndrome – Quality of Life Scale: higher results indicate worse quality of life; GTS-QOL-VAS, Visual Analogue Scale of Gilles de la Tourette Syndrome – Quality of Life Scale; higher results indicate better quality of life; doseCGI, ClinicalGlobal Impression Severity Scale; CGI-I, Clinical Global Impression –Improvement Scale; DIKJ, Depressionsinventar für Kinder und Jugendliche, German instrument to measure intensity of depression in children and adolescents; PSS-P, Perceived Stress Scale; SDQ, The Strengths and Difficulties Questionnaire; ASSF, Autismus-Spektrum Screening Fragebogen; SNAP, Swanson, Nolan and Pelham Teacher and Parent Rating; ADHD-DQ, German ADHD diagnostic questionnaire; CY-BOCS, Children’s Yale-Brown Obsessive Compulsive Disorder [cut off: <=16]; WISC, Wechsler Intelligence Scale for children- fifth edition; TMT, Trail Making Test (Trail A: Deficient: >78 sec.; Trail B: >273 sec.). CBM, cannabis based medicine.

Using the WISC, the patient achieved slightly below average scores in the domain of working memory (34%) and average results regarding processing speed (50%) (**Table 2**). To further assess cognitive performance, we also compared grades between the 3^rd^ and the 10^th^ grade (for details see **Table 3**). Overall, there was no relevant change in school marks, in particular not after initiation of CBM during the 6^th^ grade. This patient also had no symptoms of psychosis, anxiety, depression, addiction, or any other newly developed psychiatric symptoms – besides TS related mild OCD - at any time throughout the treatment period. There is no evidence for CUD and additional use of recreational cannabis was explicitly denied.

**Table 3:** Case 2: grade point averages in school before and after initiation of CBM treatment.

CBM treatment	Kind of school	Grade	Grade point average	Average
Before	Primary school	3	2.50	2.63
		4	2.75	
After	High school	5	2.63	
		6	2.58	2.74
		7	2.84	
		8	2.73	
		9	2.76	
		10	2.78	

CBM, cannabis based-medicine.

## Discussion

We present, for the first time, long-term follow-up outcomes of CBM treatment at five and six years, respectively, in two children with TS. In these two patients, long-term treatment with different THC-containing cannabinoids resulted not only in a constant improvement of tics, psychiatric comorbidities, and quality of life, but also did not cause severe adverse effects and in particular no psychological symptoms such as anxiety, psychosis, and substance abuse including CUD. Most importantly, neurocognitive test results during the course of therapy showed no evidence that the patients cognitive abilities had become below average. There was also no indication of behavioral abnormalities, social problems, neglection of social interests, or loss of interests, motivation, and drive. This is remarkable, since in both patients CBM treatment was initiated before puberty and doses of THC were relatively high.

Because of overall beneficial effects, parents and patients decided for continued treatment with CBM over many years. Based on self-assessments, in both children premonitory urges initially decreased after start of CBM treatment but increased at long-term follow-up. However, this can presumably be attributed to patients’ age, since children with TS often are unaware of premonitory urges and awareness increases with age (42).

There is evidence that cannabis use depending on dose, frequency, and age of onset can have negative effects on episodic and working memory, processing speed, executive functioning and may disrupt developmental processes leading to a poorer cognitive outcome (31, 43–45). In our study, overall, there was no evident impact of CBM on cognitive functioning However, working memory assessed with the WISC was slightly below average (34% for both cases). Since we did not apply the aforementioned psychological tests *before* the start of therapy, we cannot conclude whether CBM treatment actually affected patients’ working memory. However, neither patients nor parents noticed any changes in general memory performance and school performance. With respect to detrimental effects of cannabis on memory, it should be noted that all these reports were based on data in children using cannabis recreationally, but not on CBM in different patient groups supervised by physicians. There are several important differences between use of CBM compared to recreational use of cannabis from other sources. Firstly, recreational cannabis often comes from illegal sources and it is not clear what it contains and how it was produced. Accordingly, content of THC and CBD are unknown. It is also known that recreational cannabis usually has a quite high content of THC, while CBM, even if containing a higher percentage of THC, is usually more balanced with CBD and other substances to prevent side effects (so called “entourage effect”) (46, 47). On the contrary, CBM is produced in a controlled environment and its major ingredients, similarly to other medical products, are exactly determined and known. Secondly, recreational cannabis is usually smoked, which makes it more difficult to control. On the contrary, CBM is either vaporized or administered orally as oils, capsules, and mouth sprays. These differences in the form of administration are crucial for pharmacokinetics and pharmacodynamics and, as a result, efficacy and the profile of side effects. Thirdly, recreational cannabis is taken without any medical supervision which often leads to overuse, misuse, and even addiction (48–50), while, administration of CBM is per definition initiated and supervised by a treating physician, which is known to increase compliance and prevent overuse (51). Also, the treating physician can adjust the dose according to clinical efficacy and the profile of side effects (52). Furthermore, psychological factors, such as doctor-patient relationship may play a role in patients’ compliance. Fourthly, treating physicians screen for a history of psychiatric disorders - including family history - before starting treatment with CBM, since history of psychosis increases the risk of psychiatric side effects (53, 54). Finally, it has been suggested that TS may be caused by a deficiency or dysfunction in the endocannabinoid system (9), which might result in an altered side effect profile in this specific group of patients.

In both cases, at no time there was an uncontrolled or unwanted increase of CBM, inability to reduce the dose if wanted, any other symptoms suggesting the incidence of CUD, additional use of cannabis from other sources, or use of other drugs. This aspect is of particular interest in Case 1, since he suffers from comorbid ADHD, in which substance use disorder including CUD is a well-known and common comorbid condition (55). Based on the data presented there is no evidence that long-term THC exposure had a negative impact on brain development and cognitive function, since one boy’s school performance remained unchanged over years and the other one’s even improved. It should also be taken into account that other factors may have influenced patients’ academic performance such as the COVID-19 pandemic, which occurred during the period of assessment, as well as pubertal development.

Although findings are encouraging, a number of limitations should be acknowledged. Firstly, although according to YGTSS-TTS tic severity significantly improved after CBM treatment, it cannot be excluded that tic improvement is, at least in part, due to the typical waxing and waning course of tics. In addition, since on average tic maximum occurs at the age of 10-12, it is possible that tic improvement may be caused by a spontaneous tic improvement with increasing age. Furthermore, placebo effect cannot be excluded. Secondly, it is not possible to decide, whether improvement in patients’ quality of life and global impairment is primarily related to the improvement of tics or alternatively due to an improvement of comorbidities such as depression and ADHD. However, there is evidence that in patients with TS, cannabinoids improve not only tics but also psychiatric comorbidities (10, 15, 56, 57). Because of the sustained long-term effects, in our opinion, it can be excluded that self-reported beneficial effects are caused by THC-induced psychological effects such as euphoria. Thirdly, we measured cognitive performance using WISC only at last follow-up, but not at baseline before initiation of CBM treatment. Accordingly, no conclusion can be drawn on possible changes in patients’ cognitive strengths and weaknesses after treatment of CBM was initiated. Furthermore, we only measured working memory and processing speed, but did not perform a more extensive cognitive testing. Results of the only two domains assessed cannot be extrapolated to the total IQ results, as this was not assessed. Fourthly, in one case, CBM treatment was initiated, modified, and supervised by the patient’s parents who both are medical doctors. This may have an impact on the treatment outcome and the general attitude toward treatment.

In summary, we present two cases of minors with TS who started CBM treatment before puberty at the age of eight and 12 years, respectively, and continued treatment for five to six years resulting in sustained clinically relevant symptom improvement without severe adverse effects or negative impact on cognitive and academic performance. Although generalizability from our case reports of two single patients is limited, we suggest to take treatment with THC-containing drugs into consideration in severely affected and otherwise treatment refractory children and adolescents before thinking of surgical treatment using deep brain stimulation.

## Patient perspective

From the patient perspective, long-term treatment with CBM resulted in a clinically relevant improvement of tics and psychiatric comorbidities without causing severe adverse effects such as neurocognitive or behavioral problems and thus made it possible to attend school normally, make friends and pursue hobbies without any problems.

## Data Availability

The original contributions presented in the study are included in the article/supplementary material. Further inquiries can be directed to the corresponding author.
